# Non‐fermented tea consumption protects against osteoporosis among Chinese male elders using the Taiwan biobank database

**DOI:** 10.1038/s41598-022-11066-2

**Published:** 2022-05-05

**Authors:** Chiao-Lin Hsu, Wei-Lun Huang, Hung-Hui Chen, Jerry Cheng-Yen Lai

**Affiliations:** 1 Center for Health Management, Kaohsiung Veterans General Hospital, No. 386, Dazhong 1st Rd., Zuoying Dist., Kaohsiung City, 81362 Taiwan; 2 Center for Geriatrics and Gerontology, Kaohsiung Veterans General Hospital, No. 386, Dazhong 1st Rd., Zuoying Dist., Kaohsiung City, 81362 Taiwan; 3 Taipei Veterans General Hospital, Taitung Branch, No. 1000, Gengsheng Rd., Taitung City, Taitung County, Taiwan; 4 School of Nursing, College of Medicine, National Taiwan University, No. 1, Sec. 1, Ren’ai Rd., Zhongzheng Dist., Taipei City, 10051 Taiwan; 5 Department of Nursing, National Taiwan University Hospital, No. 7, Chung Shan S. Rd., Zhongzheng Dist., Taipei City, 10002 Taiwan; 6 Department of Medical Research, Taitung MacKay Memorial Hospital, 1, Lane 303, Changsha Street, Taitung City, 95054 Taiwan; 7 Master Program in Biomedicine, College of Science and Engineering, National Taitung University, No. 684, Section 1, Zhonghua Road, Taitung City, 950 Taiwan

**Keywords:** Endocrinology, Risk factors

## Abstract

Few studies compared the effects of non-fermented and fermented tea among the general population. We aimed to compare the risk of incident osteoporosis (OP) between non-fermented tea and fermented tea drinkers by this retrospective nationwide population-based analysis from the Taiwan Biobank. Participants ≥ 40 years who drink fermented tea (n = 2205) were compared with those who drink non-fermented tea (n = 1034) from 2008 to 2015 with a mean follow-up period of 3.64 years. OP was defined by T-score ≤ − 2.5. Multivariate Cox proportional hazards regression models were performed to estimate the risk of developing OP between the two groups. Separate models were used to determine the relationship between tea consumption and OP stratified by sex and age. There was a significant interaction between sex, age, and type of tea consumed. In men aged ≥ 60 years, the risk of developing OP decreased by 79% for those who drank non-fermented tea (hazard ratio, 0.21; 95% confidence level, 0.05–0.94) than those who drank fermented tea. Additionally, those with a family history of OP had a higher risk of developing osteoporosis. This study suggests that male elderly who consume non-fermented tea have a lower risk of OP. Drinking non-fermented tea, such as green tea, could be suggested, especially for those with a family history of osteoporosis.

## Introduction

Osteoporosis (OP), which is a chronic deconditioning musculoskeletal disease, occurs predominantly in postmenopausal women and men older than 50 years. The OP prevalence was higher among females older than 50 years than those among males in various countries^1–4^. Females older than 50 years had higher OP prevalence in East Asian countries, such as Taiwan (38.3%) and Korea (37%), than those in the United States (15.4%)^2–4^. When compared with the United States (4.3%) and Korea (7.8%), Taiwan had a much higher OP prevalence among men older than 50 years (23.9%)^2–4^. An important sequel to OP is the possibility of reduced bone mass, weakened bone quality, impaired bone flexibility, and a higher risk of fragility fractures. Fragility fractures can incur tremendous economic burden and personal socioeconomic burden on the health system^1,5^.

Previous studies have identified various risk factors for OP, such as aging, low body weight, metabolic disorders (e.g., diabetes mellitus), inflammatory diseases (e.g., rheumatoid arthritis), ankylosing spondylitis, and related medication side effects^6,7^, and researchers have further explored the influence of lifestyle behaviors on OP. The common lifestyle factors included low calcium intake, vitamin D deficiency, lack of exercise, excessive alcohol drinking, and smoking, which have also been reported in correlation with OP^8,9^. For the past two decades, tea consumption has been emphasized in studies on OP. Meta-analyses have shown that tea consumption has a positive association with bone mineral density (BMD) and a protective effect against OP^10,11^.

Tea can be categorized as fermented and non-fermented. Consumption of non-fermented tea is common in the Chinese population compared with Western countries. Given its antioxidant effects, non-fermented tea, such as green tea, is rich in flavonoids, catechins, and polyphenols that can alleviate bone degradation process^12–14^. However, fermentation reduces the content of such compounds in tea; as such, the bone protection effect of fermented tea is reduced compared with that of non-fermented tea. This has demonstrated a wide range of health benefits, including bone health^15^. By contrast, fermented tea, such as black tea or oolong tea, has a higher caffeine concentration than non-fermented tea^16^. Caffeine can increase urinary calcium excretion and bone loss^17,18^. The different components of the two may lead to their different influences on OP.

Despite many published studies on the beneficial effect of tea on bone health^10,11^, conflicting evidences were reported between two currently available clinical trials on green tea extracts. The 6-month randomized clinical trial intervention conducted by Shen et al. reported significant benefits of green tea polyphenols on bone health by muscle strength improvement in 171 postmenopausal osteopenia women^19^. On the contrary, in the 12-month Minnesota Green Tea randomized trial, there were no significant differences in BMD or adiposity after taking one-year of supplementation of green tea extract in overweight/obese postmenopausal women^20^. In addition, only few examined the effect of consuming fermented and non-fermented tea on the overall OP status among habitual tea drinkers. A recent cross-sectional study from China indicated that elderly with green tea drinking was less likely to have OP than those with non-tea drinking^21^. Another Chinese study showed that among postmenopausal women, oolong tea drinkers had higher calcaneus BMD compared with non-tea drinkers^22^. Likewise, Oolong tea drinkers exhibited higher calcaneus BMD than green or black tea drinkers among Chinese women^23^. However, a case–control study reported the effect of green or black tea drinking against osteoporotic fracture but was only statistically significant among men^24^. Additionally, other non-fermented tea, such as Yerba Mate, which is popular in South America, was associated with higher BMD in postmenopausal women^25^. Based on the abovementioned studies, consumption of various types of tea may have different effects on BMD or osteoporotic fracture, and the effects may differ by sex or age. To the best of our knowledge, no study has directly compared BMD or OP between drinkers of fermented and non-fermented tea.

Given the significantly higher prevalence of OP in Asian countries than Western countries, identification of the potential influencing factors of OP and developing preventive strategies for individuals with increased risk of OP and fragility fracture in Asians are essential^26^. Hence, the present study aimed to perform a nationwide population-based analysis comparing the risks of incident OP between non-fermented and fermented tea drinkers. Stratified analyses on age and sex were performed to identify possible determinant factors and the incidence of OP among community-dwelling population in Taiwan.

## Methods

### Data sources

By combining genetic and medical information, Taiwan Biobank (TWB) performed large-scale cohort and case–control studies on local diseases. This cohort study was a sub-dataset of nationwide TWB, which recruited 200,000 volunteers between 30 and 70 years of age without a history of cancer. De-identified information for community volunteers in the TWB was available for official research application since September 1, 2014. TWB set up an “Examination and Digital Data Release Management System” for inquiry and application services. The data were approved and in compliance with the administrative regulations on the establishment of Human Biobank. To protect the confidentiality of survey respondents, the TWB dataset was released as de-identified secondary data. The need for informed consent was waived by the IRB of the Kaohsiung Veterans General Hospital (KSVGH20-CT10-11).

### Study population

**Figure 1:**
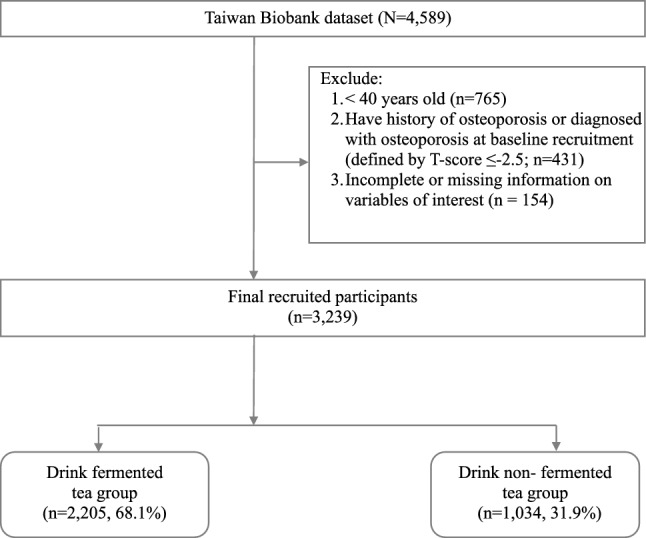
A flowchart demonstrating the enrollment of the study cohort. A total of 3239 participants had tea drinking habits, among whom 2205 (68.1%) participants had a fermented tea drinking habits and 1034 (31.9%) participants had non-fermented tea drinking habits.

A total of 4589 respondents who were habitual tea drinkers were identified from 2008 to 2015 with a mean follow-up period of 3.64 years. We excluded respondents aged less than 40 years (n = 765) and diagnosed with OP at baseline. After excluding those with OP at baseline as defined by T-score of less than − 2.5 (n = 431) and incomplete or missing information on independent variables of interests (n = 154), a total of 3239 respondents aged 40 years or more were included in the study cohort. Participants who habitually drink non-fermented and fermented tea were included in the non-fermented tea group (n = 1034; 31.9%) and the fermented tea group (n = 2205; 68.1%), respectively (Fig. 1).

### Measurements

The study variables included demographic characteristics of the participants (age, sex, residential urbanicity, and education level), body composition profile (waist circumference, hip circumference, and body mass index (BMI)), menopausal status, lifestyle behaviors (habitual tea and coffee consumption, regular diet, smoking and regular exercise status, average sleep duration, and weekday and weekend sleeping time before and after midnight), past medical history (family history of OP, diabetes mellitus (DM), and coronary heart disease), BMD profile [Baseline Z-Score and T-score]), and OP. Residential urbanicity stratification was classified into urban, suburban, and rural^27^. The BMD profile was determined by quantitative ultrasound (QUS). For community-dwelling people, QUS is a practical, easily performed, convenient, and less harmful screening tool. The habitual tea and coffee consumptions were determined by enquiring the type and average number of drinks consumed per day, week, or month. Habitual coffee consumption was enquired as “do you drink coffee on a daily basis (at least three times daily),” followed by enquiring the type and average number of drinks consumed per day, week, or month. Habitual tea consumption was enquired as “do you drink tea (loose-leaf tea, excluding floral tea) on a daily basis (at least once daily),” followed by enquiring the type and average number of drinks consumed per day, week, or month. The types of tea consumed were classified into non-fermented and fermented. Fermented tea includes back tea, Darjeeling, Assam, Qimen, Java, Kenya, oolong, baozhong, dongding, tieguanyin, jinxuan, narcissus, and baihao oolong, whereas non-fermented tea includes green tea, longjing, sencha, matcha, gyokuro, biluochun, white peony, and baihao yinzhen. The amount of tea consumption was converted to the number of cups of tea by using 200 mL as the volume of a cup of tea. Drinking tea at least once a day and drinking coffee at least three times per week were regarded as having a habitual tea and coffee consumption, respectively. Exercising at least three times per week with at least 30 min each time was determined as regular exercise. According to the World Health Organization definition, the primary outcome of the study was constructed as a binary variable for the presence or absence of OP as defined by T-score less than or equal to − 2.5.

### Statistical analysis

The participant characteristics between non-fermented and fermented tea drinkers were described by mean (standard deviation [SD]) and frequency (%) for continuous and categorical variables, respectively. The continuous, time-to-event outcome was described by median and interquartile range (IQR). The differences in baseline characteristics in different groups were compared using Student’s t-test and Chi-square test for continuous and categorical variables, respectively. Multivariate Cox proportional hazard models were performed to estimate the adjusted hazard ratios (aHR) for the risk of OP in men and women who drank tea, with adjustment for baseline demographic characteristics, lifestyle behaviors, and medical history. The interactive association among “sex and tea drinking,” “age and tea drinking,” and “sex, age, and tea drinking” with OP was examined separately. According to the potential interactions associated with tea-drinking behavior, the participant sex based on age 60 years or older was used as the stratification variable in the Cox proportional hazards regression model. Multivariate Cox proportional hazards regression models were developed for each sub-cohort. The cumulative incidences of OP in men and women who drank non-fermented and fermented tea were performed by the Kaplan–Meier product-limit method, and the difference between these two groups was compared with log-rank test. The data were analyzed using the SAS statistical software for Windows (Version 9.4; SAS Institute, Cary, NC, USA). All reported *p*-values were 2-tailed analyses with less than 0.05 level of statistical significance.

## Results

### Baseline characteristics

Table 1 compares the demographic, behavioral, and clinical variables between the groups of participants who drink non-fermented tea (n = 1034) and fermented tea (n = 2205). Similar proportion of both sexes participated in this study. Most of the participants preferred fermented tea, were aged 50 and 59 years, resided in urban areas, and attended high school. Approximately 5.5% of participants had a future OP (n = 179) within the study period, with a median follow-up period of 3.64 (3.60–3.68) years.

**Table 1:** Characteristics of the drink fermented tea and non-fermented tea groups (N = 3239).

	Total (N = 3239)	Drink fermented tea group (n = 2205; 68.1%)	Drink non-fermented tea group (n = 1034; 31.9%)	*p* value^c^
Follow-up years (95% CI)	3.64 (3.60–3.68)	3.64 (3.59–3.69)	3.64 (3.56–3.71)	0.919

Variables	Mean (SD)	Mean (SD)	Mean (SD)	
Age (year)	54.0 (7.7)	54.4 (7.7)	53.0 (7.6)	< 0.001***
**Body composition profile**				
Waist circumference	85.3 (9.4)	85.5 (9.3)	84.7 (9.5)	0.020*
Hip circumference	96.4 (6.5)	96.3 (6.3)	96.7 (6.8)	0.195
BMI	24.7 (3.4)	24.7 (3.3)	24.8 (3.5)	0.752
**BMD profile**				
Z-Score (baseline)	1.2 (1.3)	1.2 (1.3)	1.3 (1.4)	0.091
T-Score (baseline)	− 0.4 (1.4)	− 0.4 (1.4)	− 0.2 (1.4)	< 0.001***
Sleeping duration (hour)	6.8 (1.1)	6.8 (1.1)	6.8 (1.1)	0.312

	n (%)	n (%)	n (%)	
**Sex**				< 0.001***
Female	1653 (51.0)	1024 (46.4)	629 (60.8)	
Male	1586 (49.0)	1181 (53.6)	405 (39.2)	
**Age**				< 0.001***
40–49 y	986 (30.4)	615 (27.9)	371 (35.9)	
50–59 y	1370 (42.3)	950 (43.1)	420 (40.6)	
60–69 y	867 (26.8)	625 (28.3)	242 (23.4)	
≥ 70 y	16 (0.5)	15 (0.7)	1 (0.1)	
**Educational level**				0.257
College/graduate school	1445 (44.6)	1005 (45.6)	440 (42.6)	
High school	1556 (48.0)	1043 (47.3)	513 (49.6)	
None/elementary school	238 (7.3)	157 (7.1)	81 (7.8)	
**Residential urbanicity**^**a**^				0.290
Urban	1894 (58.5)	1275 (57.8)	619 (59.9)	
Suburban	1077 (33.3)	737 (33.4)	340 (32.9)	
Rural	268 (8.3)	193 (8.8)	75 (7.3)	
**Family history of osteoporosis**				0.371
No	2784 (86.0)	1887 (85.6)	897 (86.8)	
Yes	455 (14.0)	318 (14.4)	137 (13.2)	
**Diabetes mellitus**				0.754
No	2986 (92.2)	2035 (92.3)	951 (92.0)	
Yes	253 (7.8)	170 (7.7)	83 (8.0)	
**Coronary heart disease**				0.547
No	3158 (97.5)	2147 (97.4)	1011 (97.8)	
Yes	81 (2.5)	58 (2.6)	23 (2.2)	
**Total tea drinking amount**^**b**^				0.410
1–3 cup/day	1977 (61.0)	1329 (60.3)	648 (62.7)	
4–6 cups/day	777 (24.0)	537 (24.4)	240 (23.2)	
> 6 cups/day	485 (15.0)	339 (15.4)	146 (14.1)	
**Coffee drinking habits**				0.031*
No	1765(54.5)	1230 (45.8)	535 (51.7)	
Yes	1474 (45.5)	975 (44.2)	499 (48.3)	
**Regular diet**				0.047*
1 meal/day	27 (0.8)	16 (0.7)	11 (1.1)	
2 meals/day	468 (14.4)	295 (13.4)	173 (16.7)	
3 meals/day	2719 (83.9)	1875 (85.0)	844 (81.6)	
4 meals/day	21 (0.6)	17 (0.8)	4 (0.4)	
5 meals/day	4 (0.1)	2 (0.1)	2 (0.2)	
**Regular exercise**				0.002**
No	1529 (47.2)	1000 (45.4)	529 (51.2)	
Yes	1710 (52.8)	1205 (54.6)	505 (48.8)	
**Smoking experience**				< 0.001***
No	2063 (63.7)	1346 (61.0)	717 (69.3)	
Yes	1176 (36.3)	859 (39.0)	317 (30.7)	
**Weekday sleeping time**				0.073
Before midnight	2536 (78.3)	1746 (79.2)	790 (76.4)	
After midnight	703 (21.7)	459 (20.8)	244 (23.6)	
**Weekend sleeping time**				0.069
Before midnight	2473 (76.4)	1704 (77.3)	769 (74.4)	
After midnight	766 (23.6)	501 (22.7)	265 (25.6)	
**T-score** ≤ − **2.5 (outcome)**				0.144
No	3060 (94.5)	2092 (94.9)	968 (93.6)	
Yes	179 (5.5)	113 (5.1)	66 (6.4)	

CI, confidence interval; mon, month; SD, standard deviation; BMI, body mass index; BMD, bone mineral density.

*p* < 0.05*; *p* < 0.01**; *p* < 0.001***.

^a^Residential urbanicity stratification was classified into urban, suburban, and rural according to the population density, ratio of educated people, age (aged ≥ 65 years), farmers, and the number of physicians per 100,000 people as defined by Liu et al.^27^.

^b^Total tea drinking amount was divided by cups (200 ml).

^c^Unadjusted *p*-value (Chi-square test or Student’s t-test).

### Effects of non-fermented tea consumption on the risk of OP

Predictors for OP are presented in Table 2. The association between non-fermented tea consumption and the risk of developing OP was not significant; however, age, sex, educational level, and BMI were significantly associated with OP (Model 1). Participants who were female (aHR, 1.50; 95% CI 1.00–2.27; *p* = 0.051, borderline statistical significance), aged 60 years or older (aHR, 2.57; 95% CI 1.85–3.58; *p* < 0.001), with high school (aHR, 1.54; 95% CI 1.11–2.14; *p* = 0.01), and lower BMI (aHR, 0.88; 95% CI 0.80–0.96; *p* = 0.006) had a higher risk of developing OP. Although the risk of developing OP was lower among drinkers of fermented tea than non-fermented tea (5.1% vs. 6.4%; Table 1), the difference was not significant after adjusting for baseline demographic characteristics of the participants, lifestyle behaviors, and past medical history. The interactions between sex and tea drinking (Model 2), age and tea drinking (Model 3), and sex, age, and tea drinking (Model 4) on OP were significant (Table 2).

**Table 2:** Multivariate Cox proportional hazards regression model for osteoporosis.

Variables	Adjusted HR (95% CI)	*p *value
**Model 1**^**b**^		
Sex: Female (vs Male)	1.50 (1.00–2.27)	0.051
Age: ≥ 60 y (vs 40 ~ < 60 y)	2.57 (1.85–3.58)	< 0.001***
Educational level (vs College or graduate school)		
High school	1.54 (1.11–2.14)	0.010 **
None or elementary school	1.36 (0.79–2.34)	0.262
Residential urbanicity (vs Urban)		
Suburban	0.79 (0.57–1.09)	0.147
Rural	0.69 (0.38–1.26)	0.225
Baseline comorbidity		
Family history of osteoporosis: Yes (vs No)	1.31 (0.87–1.96)	0.192
Diabetes mellitus: Yes (vs No)	1.35 (0.83–2.19)	0.229
Coronary heart disease: Yes (vs No)	0.86 (0.35–2.13)	0.742
Body Composition profile		
Waist circumference	1.02 (0.99–1.05)	0.304
Hip circumference	1.03 (0.98–1.07)	0.216
BMI	0.88 (0.80–0.96)	0.006**
Behavioral factors		
Tea drinking: Non-fermented tea (vs Fermented tea)	1.22 (0.89–1.66)	0.213
Total tea drinking amount^a^ (vs 1–3 cup/day)		
4–6 cups/day	1.22 (0.86–1.74)	0.269
> 6 cups/day	1.20 (0.78–1.85)	0.417
Coffee drinking habits: Drink coffee (vs No)	1.11 (0.82–1.51)	0.482
Regular diet: > 3 meals per day (vs 1–3 meals/day)	1.15 (0.28–4.69)	0.850
Regular exercise: Yes (vs No)	0.74 (0.54–1.01)	0.056
Smoking experience: Yes (vs No)	1.03 (0.68–1.56)	0.883
Weekday Sleeping Time: After midnight (vs Before midnight)	0.81 (0.32–2.05)	0.657
Weekend Sleeping Time: After midnight (vs Before midnight)	0.84 (0.34–2.07)	0.700
**Model 2**^**c**^		
Educational level (vs College or graduate school)		
High school	1.54 (1.11–2.14)	0.010*
None or elementary school	1.37 (0.80–2.35)	0.259
BMI	0.88 (0.80–0.96)	0.006**
Sex and tea drinking		
Male, drink fermented tea (n = 1181)	1	
Male, drink non-fermented tea (n = 405)	1.08 (0.64–1.83)	0.775
Female, drink fermented tea (n = 1024)	1.42 (0.89–2.25)	0.139
Female, drink non-fermented tea (n = 629)	1.84 (1.13–3.00)	0.014*
**Model 3**^**d**^		
Educational level (vs College or graduate school)		
High school	1.55 (1.11–2.15)	0.010**
None or elementary school	1.34 (0.78–2.31)	0.285
BMI	0.88 (0.80–0.97)	0.008**
Age and tea drinking		
40 ~ < 60 Y, drink fermented tea (n = 1565)	1	
40 ~ < 60 Y, drink non-fermented tea (n = 791)	1.52 (1.02–2.26)	0.041*
≥ 60 Y, drink fermented tea (n = 640)	3.14 (2.10–4.69)	< 0.001***
≥ 60 Y, drink non-fermented tea (n = 243)	2.77 (1.66–4.60)	< 0.001***
**Model 4**^**e**^		
Educational level (vs College or graduate school)		
High school	1.51 (1.09–2.10)	0.014*
None or elementary school	1.30 (0.76–2.25)	0.339
BMI	0.88 (0.80–0.97)	0.008**
Regular exercise: Yes (vs No)	0.73 (0.53–0.99)	0.044*
Sex, age and tea drinking		
Male, 40 ~ < 60 Y, drink fermented tea (n = 785)	0.22 (0.11–0.42)	< 0.001***
Male, 40 ~ < 60 Y, drink non-fermented tea (n = 294)	0.35 (0.17–0.74)	0.005**
Male, ≥ 60 Y, drink fermented tea (n = 396)	0.45 (0.23–0.87)	0.017*
Male, ≥ 60 Y, drink non-fermented tea (n = 111)	0.12 (0.03–0.53)	0.005**
Female, 40 ~ < 60 Y, drink fermented tea (n = 780)	0.21 (0.11–0.37)	< 0.001***
Female, 40 ~ < 60 Y, drink non-fermented tea (n = 497)	0.33 (0.18–0.60)	< 0.001***
Female, ≥ 60 Y, drink fermented tea (n = 244)	0.96 (0.54–1.69)	0.885
Female, ≥ 60 Y, drink non-fermented tea (n = 132)	1	

CI, confidence interval; HR, hazard ratio; BMI, body mass index.

*p* < 0.05*; *p* < 0.01**; *p* < 0.001***.

^a^Total tea drinking amount was divided by cups (200 ml).

^b^Hazard ratio without adjusting confounding factors.

^c^Hazard ratio adjusted for age, residential urbanicity, family history of osteoporosis, diabetes mellitus, coronary heart disease, waist circumference, hip circumference, total tea drinking amount, coffee drinking habits, regular diet > 3 meals per day, regular exercise, smoking experience, weekday sleeping time, and weekend sleeping time.

^d^Hazard ratio adjusted for sex and all variables in footnote c, except for age.

^e^Hazard ratio adjusted for all variables in footnote c, except for age and sex.

### Effect of non-fermented tea consumption on the development of osteoporosis, stratified by sex

Table 3 shows that regardless of sex, there was no significant difference between fermented and non-fermented tea consumption on the increased risk of OP. After covariate adjustment, men with a family history of OP were associated with an increased risk of OP (aHR, 2.00; 95% CI 1.11–3.59). By contrast, women aged 60 years or older (aHR 3.11; 95% CI 2.04–4.76), with postmenopausal status (aHR, 3.21; 95% CI 1.43–7.19), and consumed more than six cups of tea daily compared with one to three cups daily were associated with increased risk of OP (aHR, 1.83; 95% CI 1.04–3.20). Overall, women with a higher BMI had a decreased risk of OP (aHR, 0.88; 95% CI 0.78–0.99); however, the association was not significant in men.

**Table 3:** Multivariate Cox proportional hazards regression model for osteoporosis by sex and age.

Variables	Men			Women		
	≥ 40 Y	40 ~ < 60 Y	≥ 60 Y	≥ 40 Y	40 ~ < 60 Y	≥ 60 Y
	HR (95% CI)^b^	HR (95% CI)^c^	HR (95% CI)^b^	HR (95% CI)^b^	HR (95% CI)^d^	HR (95% CI)^c^
**Age**						
40 ~ < 60 y	1	-	-	1	-	-
> = 60 y	1.48 (0.86–2.52)	-	-	3.11 (2.04–4.76)***	-	-
**Menopausal status**						
Pre-menopausal	-	-	-	1	1	-
Post-menopausal	-	-	-	3.21 (1.43–7.19)**	3.13 (1.37–7.14)**	-
**Family history of OP**						
No	1	1	1	1	1	1
Yes	2.00 (1.11–3.59)*	1.46 (0.66–3.22)	5.22 (2.00–13.66)***	1.04 (0.59–1.86)	1.72 (0.85–3.51)	0.51 (0.18–1.48)
BMI	0.87 (0.74–1.03)	0.79 (0.64–0.98)*	1.09 (0.84–1.43)	0.88 (0.78–0.99)*	0.88 (0.74–1.04)	0.87 (0.74–1.03)
**Tea drinking**						
Fermented tea	1	1	1	1	1	1
Non-fermented tea	1.02 (0.60–1.73)	1.42 (0.76–2.66)	0.21 (0.05–0.94)*	1.30 (0.88–1.92)	1.58 (0.92–2.73)	1.07 (0.59–1.95)
**Total tea drinking amount**^**a**^						
1–3 cup/day	1	1	1	1	1	1
4–6 cups/day	1.33 (0.77–2.29)	1.81 (0.93–3.52)	0.53 (0.18–1.55)	1.14 (0.71–1.84)	1.21 (0.60–2.43)	1.17 (0.60–2.29)
> 6 cups/day	0.83 (0.42–1.66)	0.76 (0.30–1.92)	0.73 (0.25–2.14)	1.83 (1.04–3.20)*	2.49 (1.19–5.23)*	1.39 (0.56–3.49)

Y, years old; CI, confidence interval; HR, hazard ratio; OP, osteoporosis; BMI, body mass index.

*p* < 0.05*; *p* < 0.01**; *p* < 0.001***.

^a^Total tea drinking amount was divided by cups (200 ml).

^b^Hazard ratio adjusted for educational level, residential urbanicity, diabetes mellitus, coronary heart disease, waist circumference, hip circumference, coffee drinking habits, regular diet > 3 meals per day, regular exercise, smoking experience, weekday sleeping time, and weekend sleeping time.

^c^Hazard ratio adjusted for all variables in footnote b, except for the regular diet variable.

^d^Hazard ratio adjusted for all variables in footnote b, except for the status of coronary heart disease.

### Effect of non-fermented tea consumption on the development of osteoporosis, stratified by sex and age

The non-fermented tea group had a 79% lower risk of developing OP (aHR, 0.21; 95% CI 0.05–0.94) in older men than in men younger than 60 years, whereas an increased risk of OP was not observed in female tea drinkers of any age (Table 3). Thus, non-fermented tea drinkers were protected against OP among men aged 60 years and older. Older men with a family history of OP had a 322% higher risk of OP than in men before age stratification (aHR, 5.22; 95% CI 2.00–13.66 vs. aHR, 2.00; 95% CI 1.11–3.59). After age stratification, a higher BMI was no longer associated with a decreased risk of OP in women. By contrast, younger men with a higher BMI were likely to be associated with a decreased risk of developing OP (aHR, 0.79; 95% CI 0.64–0.98). In women younger than 60 years, those with postmenopausal status had increased the risk of OP (aHR, 3.13; 95% CI 1.37–7.14), similar in magnitude to that of the study cohort before stratification (aHR, 3.21; 95% CI 1.43–7.19), which suggested that the outcome was independent of women’s age. After adjusting for potential confounders, drinking more than six cups of tea per day was no longer a significant predictor of OP in older women. Younger women who consumed six cups of tea per day were associated with a 66% higher risk of OP compared with women before age stratification (aHR, 2.49; 95% CI 1.19–5.23 vs. aHR, 1.83; 95% CI 1.04–3.20).

**Figure 2:**
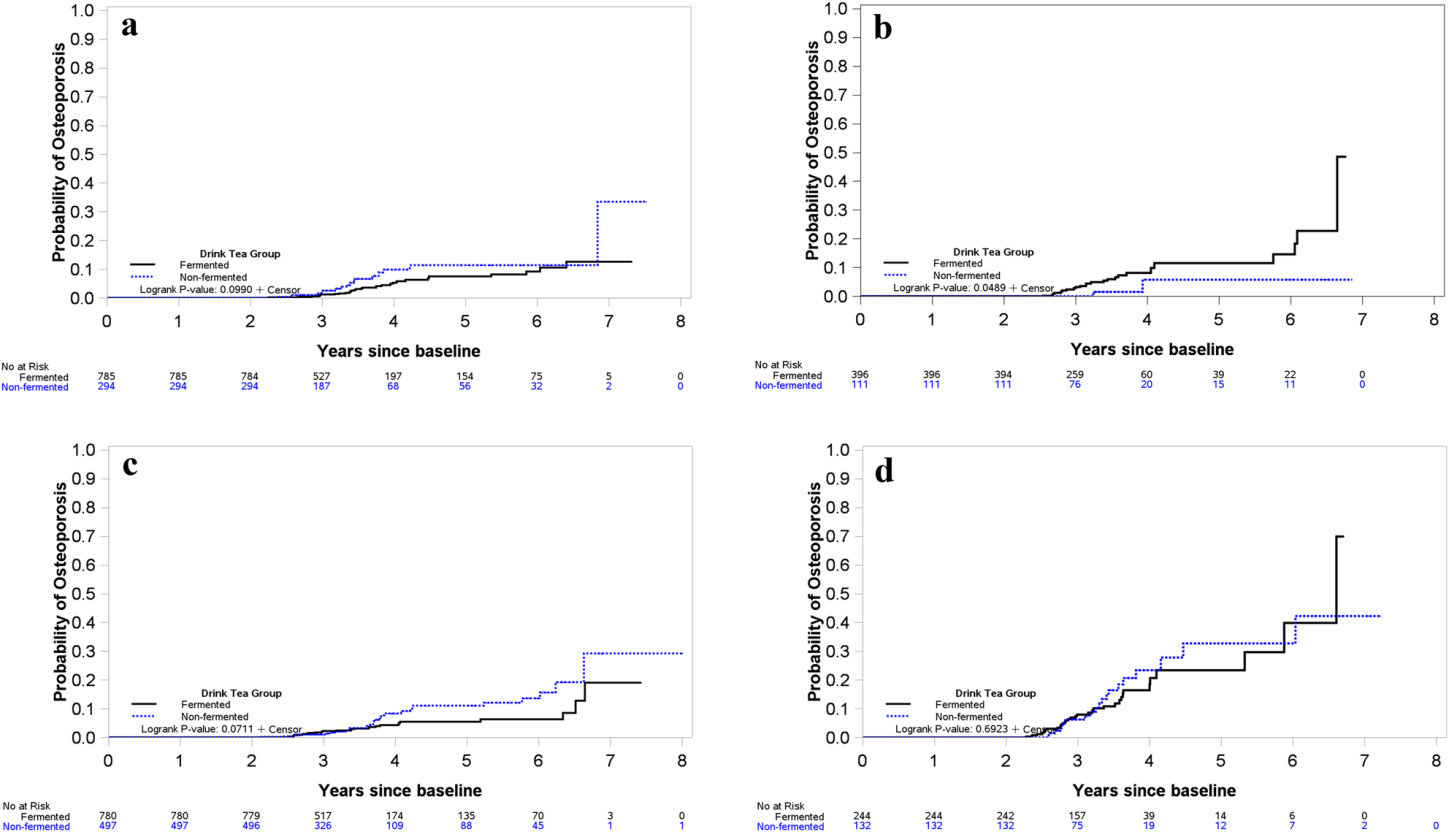
The cumulative incidences of osteoporosis in men (**a** younger than 60 years; **b** older than 60 years) and women (**c** younger than 60 years; **d** older than 60 years) who drank non-fermented tea and fermented tea were performed by the Kaplan–Meier product-limit method. Protective effect against osteoporosis were seen only in non-fermented tea drinkers who were male and aged 60 years or more, and the difference between these two groups was compared with log-rank test (*p* < 0.05).

The Kaplan–Meier curves of cumulative incidence of OP was significantly lower in the non-fermented tea group than in the fermented tea group over the entire 8 years of observation among men aged 60 years or older (log-rank test, *p* = 0.049; Fig. 2c). No significant difference among women younger or older than 60 years and men aged younger than 60 years old (Fig. 2a, b, d).

## Discussion

Our nationwide population-based study suggested that female sex and age of 60 years or older were the significant predictors of OP in Taiwan. In addition, the interaction between sex, age, and type of tea consumed was significant. This result proved that associations between type of tea drinking and OP differed in terms of sex and age. Furthermore, male elderly with non-fermented tea drinking behavior had a lower risk of OP. Much evidence from animal and epidemiological studies links non-fermented tea and bone health due to the antioxidant effects of flavonoids, catechins, and polyphenols^12,13^. Despite the similar preference of both sexes for non-fermented tea (12.5–19.4%) and fermented tea (31.6–36.5%) in our study, a 79% decreased risk of OP was found only in older men during the follow-up period, in which non-fermented tea consumption had a protective (or risk-reduction) effect on bone loss. Additionally, high school education and lower BMI were the important predictors of OP. Higher prevalence of OP was observed in men with a family history of OP and in women with advanced age, postmenopausal status, total daily tea consumption of over six cups, and lower BMI. Unlike the published literatures that focus on the characteristics between tea and non-tea drinkers, this study further adds the findings of OP comparison between drinkers of fermented and non-fermented tea to the literature.

The relationship between types of tea drinking and OP may differ by sex and age. Our study results identified that male elderly with non-fermented tea drinking behavior were less likely to have OP than those who had fermented tea drinking behavior, which was in accordance with a Chinese case–control study by Huang and Tang^24^. On the contrary, Li et al.^23^ reported the positive association with BMD measures in adult women only. Another study from Argentina found that postmenopausal women drinking Yerba Mate, a type of non-fermented tea, were more likely to have higher BMD^25^. However, it is important to realize the wide disparities in study designs and selected populations between studies, which limits the direct comparison of our study results to the available literature.

In contrast to the OP prevalence in Taiwanese men (23.9%) and women (38.3%) older than 50 years^4^, we found an overall lower risk of OP (5.5%) among our study participants aged older than 40 years with tea drinking behaviors. This may be partially explained by the presence of catechins, much higher in non-fermented than fermented tea types. Catechins constitutes more than 80% of the polyphenols in (non-fermented) green tea, which promotes bone-forming osteoblastic activities in rat osteoblast-like osteosarcoma cells (UMR-106) and inhibits bone-resorpting osteoclast differentiation using mouse macrophage cells (RAW 264.7) ^28,29^. The osteogenesis promotion and the inhibition of adipocyte formation in both human and rat mesenchymal stem cells also supported the potential use of green tea polyphenols against disease such as osteoporosis^30,31^. Shen et al. found that polyphenols mitigated bone loss and, at higher doses, suppressed bone turnover in the trabecular and cortical bone in animal rat models^32–34^. The lack of the protective effect of non-fermented tea drinking behavior in female elderly can be explained by the reduction in estrogen level during postmenopausal status, which counteract the protective effect of the catechins. Moreover, men elderly had a much lower prevalence of osteoporosis and related fractures than female elderly. In conjunction with the much higher proportion of men elderly having daily consumption of more than six cups of non-fermented tea than female elderly (17.1 versus 8.3%) in our study population, the protective effect of non-fermented tea can be partially explained herein and is expected to be more pronounced in men elderly than in female elderly aged 60 years or more. Nonetheless, further research is needed to investigate the mechanisms underlying the protective effect in male elderly with non-fermented tea drinking behavior, and a complex interaction among hormonal, genetic and behavioral factors might be involved.

Advanced age and postmenopausal status are clinically important risk factors for OP in women^35,36^. Peak bone mass is usually achieved approximately at an age of 35 and remains relatively constant until entering menopause^37^. Menopause contributes to gonadal degeneration and reduces circulating estrogen over several years, leading to subsequent deterioration of BMD^8^. Since the starting age of menopause varies considerably, fully isolating the effect of advanced age and menopausal status on OP is difficult. The reduction in estrogen level during menopause contributes to OP and hip fracture^38–40^. Hip fracture is an important cause of death in older women.

Daily consumption of more than six cups of tea (> 1200 mL) compared with one to two cups of tea (200–400 mL) showed a significant correlation with higher risk of OP in younger female participants, possibly due to the excessive fluoride and caffeine-related bone loss^41,42^. However, heavy tea consumption did not show a negative effect on male participants. This may contribute to sex differences in caffeine metabolism^31^ and hormone interaction disparity. Additionally, larger bone size and lean muscle mass in men may compromise the adverse effects of overt caffeine^43^.

Women with lower BMI had an increased risk of OP. After covariate adjustment, this decreased by 12% for each unit increase in BMI, confirming the finding reported by Lloyd et al.^44^ and one meta-analysis^45^. A higher BMI with larger muscle mass or fat mass imposed a greater gravitational load on bone, leading to an increased BMD to accommodate this load. The increased body fat could act as an important estrogen source for the production of estrogen and other hormones that are involved in the osteoblast and osteoclast activity and facilitated the development of bone mass^46^. To reduce the risk of OP, women should be advised to avoid being underweight.

Those with a family history of OP have a genetic predisposition toward developing OP^47^. Although older women had a higher proportion of OP family history than older men (11.4% > 7.9%), we found an increased risk of OP among older men with a family history of OP but did not reach statistically significant among older women possibly due to the interaction between family history of OP and postmenopausal status. The low estrogen level after menopause significantly increases the risk of developing OP and OP-related fractures, which are more destructive to bones than a family history of OP. However, we cannot underestimate its associated risk. Hence, elderly women with family history of OP are encouraged to adhere to a healthy lifestyle and OP screening test on a routine basis.

Participants with lower education level had a higher risk of OP in our study. Earlier research reported an association between higher education level with reduced risk of OP compared with those with lower education level^48^. Education level may provide “protection” against OP as higher education level is associated with better healthcare knowledge, which can promote more health-seeking lifestyles and behaviors against OP.

## Strengths and limitations

Being a nationwide population-based dataset with a relatively large representative sample size, the major strength of the use of TWB was the enhancement of external validity of the current findings. However, it has several limitations. First, OP evaluation with QUS rather than dual-energy X-ray absorptiometry screening may underestimate the incidence of OP. Nevertheless, this is sufficient for the early detection and prevention in community screening. Second, the data of tea and coffee consumed were self-reported, which may not exclude a recall bias and response bias. Potential confounders that may be associated with OP, such as calcium or vitamin D intake, were not included in this study. Third, our data do not comprise past history of autoimmune disease, hypogonadism, previous fracture, and rheumatoid arthritis, which may confound our results. Although rheumatoid arthritis is an important risk factor for OP, it has a minor confounding effect due to its low incidence rate of 15.8 cases per 100,000 population^49^. Fourth, the current medication therapy and the details of tea drinking (e.g., the species of tea, the processing method, the components of tea product, and the amount of tea use for preparing the tea beverage) were not available in the Biobank data, which could have resulted in residual confounding. Finally, we did not assess the long-term effect of non-fermented tea consumption on OP or fragility fractures, and this issue should be further studied.

## Conclusions and implications

The present study demonstrated that OP risk increased in men with a family history of OP and in women with advanced age, postmenopausal status, total daily tea consumption of over six cups, and lower BMI. Non-fermented tea had a protective effect against OP only in men aged 60 years or more. Habitual consumption of non-fermented tea had no protective effect against OP among women and younger men, but the lack of association was probably due to the short follow-up period of 3.64 years. Given that only a few studies have investigated the relationship between consumption of non-fermented tea and OP in the Chinese population, these data may serve as a basis of comparison for future Asian studies.

## Data Availability

Not applicable.

## Abbreviations

OP: Osteoporosis
TWB: Taiwan biobank
DM: Diabetes mellitus
BMD: Bone mineral density
BMI: Body mass index
QUS: Quantitative ultrasound
mL: Millimeters
SD: Standard deviation
HR: Hazard ratio
aHR: Adjusted hazard ratio
